# A Dual-Mode Near-Infrared Optical Probe and Monte Carlo Framework for Functional Monitoring of Rheumatoid Arthritis: Addressing Diagnostic Ambiguity and Skin Tone Robustness

**DOI:** 10.3390/s26041179

**Published:** 2026-02-11

**Authors:** Parmveer Atwal, Ryley McWilliams, Ramani Ramaseshen, Farid Golnaraghi

**Affiliations:** 1 School of Mechatronic Systems Engineering, Simon Fraser University, Surrey, BC V3T 0A3, Canada; parmveer_atwal@sfu.ca (P.A.); ryley_mcwilliams@sfu.ca (R.M.); ramani_ramaseshan_2@sfu.ca (R.R.); 2 Department of Medical Physics, BC Cancer—Abbotsford, Abbotsford, BC V2S 0C2, Canada

**Keywords:** rheumatoid arthritis, near-infrared spectroscopy, Monte Carlo simulation, optical phantom, biomedical optics, equipment design, disease detection, disease grading

## Abstract

Current diagnostic modalities for rheumatoid arthritis (RA), such as Magnetic Resonance Imaging (MRI) and ultrasound (US), excel at visualizing structural pathology but are either resource-intensive or often limited to morphological assessment. In this work, we present the design and technical validation of a low-cost continuous-wave near-infrared (NIR) dual-mode optical probe for functional monitoring of joint inflammation. Unlike superficial imaging, NIR light penetrates approximately 3–5 cm and is tissue and wavelength dependent, enabling trans-illumination of the synovial volume. The system combines reflectance and transmission geometries to resolve the ambiguity between disease presence and disease severity. To validate the diagnostic logic, we employed mcxyzn Monte Carlo (MC) simulations to model the optical signature of RA progression from early onset to EULAR-OMERACT grade 2 pannus hypertrophy on a simplified finger model, based on several tissue models in the literature and supported by physical measurements on a multilayer silicone phantom and in vivo signal verification on human volunteers. Our results demonstrate a distinct functional dichotomy: reflectance geometry serves as a binary discriminator of synovial turbidity onset, while transmission flux serves as a monotonic proxy for pannus volume, exhibiting a quantifiable signal decay consistent with the Beer–Lambert law. Signal verification on a subject with confirmed RA pathology demonstrated a significant increase in the effective attenuation coefficient (${µ}_{eff}\;\sim \;0.59$ mm^−1^) compared to the healthy baseline (${µ}_{eff}\;\sim \;0.47\;$ mm^−1^). Furthermore, simulation analysis revealed a critical “metric inversion” in darker skin phenotypes (Fitzpatrick V–VI), where the standard beam-broadening signature of inflammation is artificially suppressed by epidermal absorption. We conclude that while transmission flux remains a robust grading metric across diverse skin tones, morphological beam-shape metrics are not robust, particularly in high-absorption populations. By targeting the hemodynamic precursors of structural damage, this dual-mode probe design offers a potential pathway for longitudinal, quantitative monitoring of disease activity at the point of care, while the systematic use of the Monte Carlo framework provides insight into the measurement geometry most suitable for a given clinical endpoint, whether that be detecting the presence or severity of rheumatoid arthritis.

## 1. Introduction

Rheumatoid arthritis (RA) is a chronic autoimmune disease characterized by synovial inflammation, hyperplasia, and progressive joint destruction. Healthy joints have synovial fluid within the joint capsule. Synovial fluid is a clear yellowish liquid, found to have negligible scattering and water-like absorption [1]. As RA progresses, there is increased synovitis, and the synovial fluid is replaced with tissue growth, which the immune system attacks. This grayish tissue, referred to as the pannus, extends both laterally and axially as the disease progresses [2] and has been found to have optical parameters characteristic of blood-rich soft tissue [3]. Early diagnosis and frequent monitoring are critical, as timely intervention with disease-modifying anti-rheumatic drugs (DMARDs) can prevent irreversible bone erosion [2]. While Magnetic Resonance Imaging (MRI) and ultrasound (US) are the current gold standards for detecting early inflammatory signs [4,5], such as synovitis and bone marrow edema, they face significant implementation barriers. MRI is costly, time-consuming, and often requires contrast agents, limiting its utility for routine longitudinal assessment [4,6]. Ultrasound, though more accessible, is highly operator-dependent, though this is improving [7]. Consequently, there remains an unmet need for a low-cost, quantitative, and non-invasive modality capable of objective grading at the point of care.

Near-infrared (NIR) optical sensing offers a promising alternative by probing the functional and structural properties of joint tissue. Unlike superficial imaging techniques, light in the 650–950 nm spectral window can penetrate several centimeters through soft tissue and bone, enabling full trans-illumination of the joint capsule [8]. In this region, photon transport is governed by the scattering of cellular structures and absorption by water and chromophores such as hemoglobin [7,9,10,11]. Previous studies have employed Diffuse Optical Tomography (DOT) to reconstruct 2D maps of optical coefficients [1,12]. However, full tomographic reconstruction is computationally expensive and requires complex, multi-source arrays that are often impractical for handheld clinical use.

In this work, we present a technical validation framework via Monte Carlo simulations and a simplified, dual-mode continuous-wave optical probe designed for simultaneous subsurface anomaly detection and RA severity grading up to EULAR-OMARCT grade 2 [13] in finger joints. Rather than attempting full image reconstruction, we propose a sensor-based approach, where reflectance geometry serves as a binary discriminator for disease presence and transmission geometry provides a monotonic proxy for disease severity (pannus volume). To validate this approach, we employed mcxyzn, a Cartesian Monte Carlo code to accurately model photon transport across curved tissue interfaces [14,15]. We define a comprehensive validation hierarchy: experimental reproducibility data and in vivo signal verification from volunteers were used to benchmark the model’s numerical noise floor, while simulations were used to characterize diagnostic limits across diverse skin phenotypes. By confirming that the simulated pathological signatures exceed the inherent variance of the stochastic Monte Carlo process, we provide a validated framework for interpreting the relationship between measurement geometry and diagnostic endpoints. The results demonstrate that while reflectance captures superficial scattering changes associated with the onset of inflammation, careful selection of a transmission flux metric provides a robust metric for grading synovial volume.

## 2. Materials and Methods

### 2.1. Optical Probe Design

**Figure 1:**
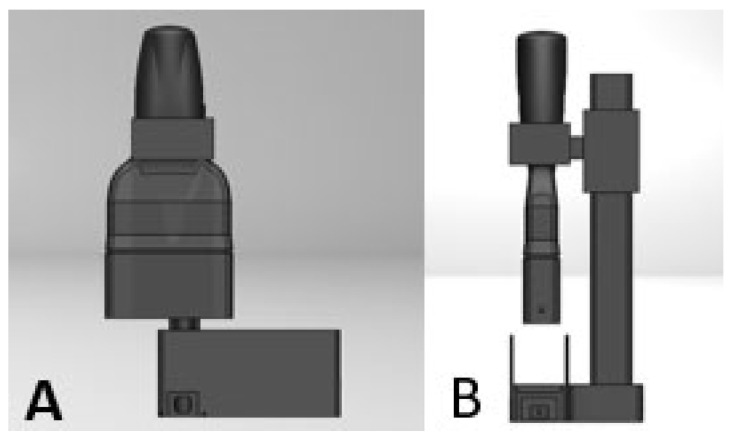
The dual-mode NIR probe: front (**A**) and side (**B**) views. The finger can be inserted in the slot at the bottom, allowing for simultaneous reflectance and transmission measurements.

The dual-mode continuous-wave near-infrared (NIR) probe, shown in Figure 1, is a modification of the latest version of the custom handheld reflectance probe designed by our lab; more detailed information on the hardware and design evolution of the handheld probe are discussed in previous work from our lab [8]. For this study the probe was modified, with one of the encapsulated light-emitting diodes (eLED) remounted to a new housing directly below the probe’s sensor, while the other eLED remained in line with the long axis of the sensor. In this geometry the probe can acquire transmission and reflectance measurements from finger joints sequentially, enabling complementary assessment of the tissue. This new design accommodates proximal interphalangeal (PIP) joints and maintains consistent source detector alignment via a vertical sliding arm. This arm also allows for accommodation of finger joints of various thickness to be measured, with full contact ensured by manual pressure on the upper component. Data acquisition was controlled through the same custom interface used for the handheld probe, allowing adjustment of wavelength selection, integration time, and real-time signal capture.

Detection was performed using a linear charge-coupled device (CCD) array (Sony ILX511, Sony Group Corporation, Sony City, Japan) [16], producing spatially resolved intensity profiles across the illuminated joint. The detector pixel pitch of 14 µm, software-binned to an effective pitch of 0.224 mm, has an effective imaging length of 28.672 mm. Both eLEDs operate at four discrete wavelengths (690, 750, 800, and 850 nm) to allow for future multispectral analysis. However, for the specific purpose of this study, characterizing the structural scattering changes associated with pannus formation, data analysis and simulations were restricted to the 850 nm channel. Wavelength selection, in general, is dependant on what is being studied, and in particular, if the concentration of constituent materials is desired from a heterogenous material, multiple wavelengths must be judiciously chosen so as to make the problem more well-posed. However, single wavelength data is still useful, as we show.

This wavelength was selected to optimize the trade-off between scattering and absorption. Optical scattering in biological tissue decreases with wavelength (${µ}_{s}^{\prime }\propto \lambda^{-b}$) [11]. Operating at 850 nm, the longest wavelength in our array prior to the onset of significant water absorption bands (>900 nm), minimizes scattering losses in the dense cortical bone, maximizing the signal-to-noise ratio for trans-illumination. Although 850 nm is slightly offset from the hemoglobin isosbestic point (~805 nm), it avoids the strong deoxy-hemoglobin absorption seen lower wavelengths. Consequently, signal attenuation remains dominated by the increasing volume of pannus formation rather than transient oxygenation changes in the joint, with reduced cross-talk from scatter.

### 2.2. Phantom Fabrication

**Figure 2:**
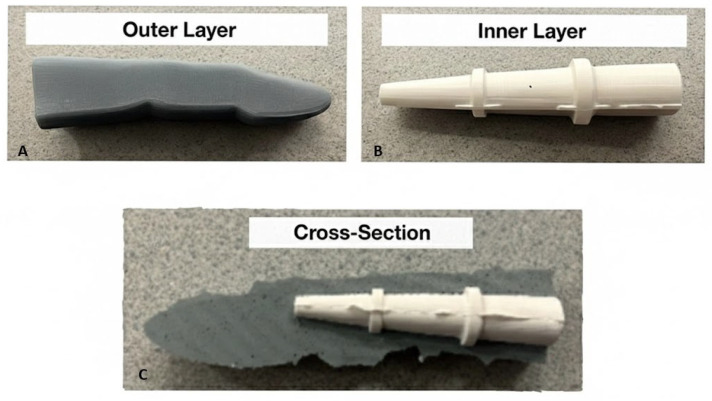
Fabrication of the multilayer silicone finger phantom. (**A**) Cured phantom showing final form and surface finish, (**B**) internal bone-like insert, and (**C**) insert embedded in gray silicone representing soft tissue.

To evaluate probe performance under controlled optical conditions, a tissue-mimicking finger phantom (see Figure 2) was fabricated following established near-infrared phantom design approaches [17,18]. The phantom geometry approximated the layered anatomy of a human finger joint, enabling systematic investigation of transmission and reflectance responses.

The phantom matrix consisted of a platinum-cured silicone elastomer (Mold Max™ 10), selected for its mechanical stability and refractive index (n ≈ 1.4), which is comparable to that of soft biological tissue. Optical scattering was introduced using titanium dioxide (TiO_2_) powder, while broadband absorption within the NIR window was achieved using carbon-black pigment.

The phantom comprised two concentric regions: an inner optically modified core representing the combined cortical bone and synovial region, and an outer layer representing skin and subcutaneous tissue. This simplified layered configuration enables controlled adjustment of the inner region’s optical properties to emulate progressive inflammatory changes associated with rheumatoid arthritis. By varying the concentrations of scatterers and absorbers within this region, the phantom provided a reproducible reference for benchmarking attenuation trends observed in simulations and in vivo measurements.

### 2.3. In Vivo Signal Verification and Feasibility Testing

Following phantom characterization, an in vivo signal verification study was conducted to benchmark the system’s noise floor in a realistic physiological environment. Thirteen healthy volunteers (ages 20–30) were recruited to serve as “living phantoms.” The objective was not clinical diagnosis, but strictly to quantify the “biological noise” inherent to live measurements—specifically, the signal variability arising from skin thickness, finger geometry, and involuntary tremor. Measurements were obtained from the index and middle fingers to establish a baseline reproducibility profile for the device.

To verify the system’s dynamic range in the presence of optical turbidity, a feasibility test was performed on a reference subject (age 72) with a confirmed history of rheumatoid arthritis affecting the proximal interphalangeal joints. This single pathological case was included solely to validate that the sensor could detect a change in the effective attenuation coefficient (${µ}_{eff}=\sqrt{3{µ}_{a}{µ}_{s}^{\prime }}$) consistent with the scattering properties modeled in our simulation.

This data collection was conducted as a Quality Assurance and Equipment Validation exercise. All participants were fully briefed on the nature of the device and provided informed consent prior to scanning. No personally identifiable information (PII) or clinical health data were recorded.

### 2.4. Computational Modelling (Using Mcxyzn Software)

**Figure 3:**
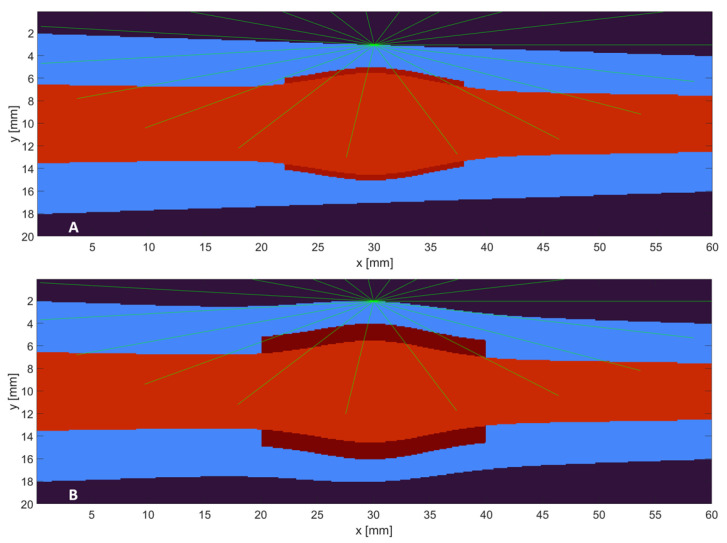
Digital models of a finger joint: (**A**) healthy and (**B**) arthritic. Blue indicates generic tissue; red indicates bone. The transformation of the synovial fluid to a thickened pannus is evident in the annulus around the knuckle. Green highlights the LED location and emitted photons.

After establishing the probe’s ability to distinguish between healthy and inflamed joints in a physical environment, we implemented numerical models of various finger joints using mcxyzn [15], a voxelized extension of the venerable MCML [14], to handle photon refraction at curved tissue interfaces accurately. MCML is made available by the Optical Medical Imaging Laboratory (OMLC) and maintained at omlc.org for public use; mcxyzn is available on GitHub at https://github.com/phongatran/mcxyzn (accessed on 7 January 2025. This digital environment enabled in-depth characterization of optical signatures associated with disease progression and provided a platform for evaluating the system’s performance under varying anatomical and optical conditions. Cross-sections of two of the finger models used are shown in Figure 3.

All simulations were performed at 850 nm, matching the probe wavelength. Each tissue was assigned optical properties. Determining optical properties of materials is an ongoing area of research, and it is difficult to find consensus for the absorption and scattering coefficients, ${µ}_{a}$ and ${µ}_{s}^{\prime }$, respectively, which are key parameters characterizing NIR light behaviour in various tissues. As such, select publications were chosen from which to choose representative models. The interested reader is encouraged to begin with the comprehensive review by Jacques [11].

The absorption coefficient for bone was modified from [19], wherein dry bovine bone matrix was characterized and incorporated into the model, using the improved lipid spectrum from [20]. The reduced scattering coefficient was taken from [21], wherein the coefficient was modelled in part on results from [19], as well as other works. These results are also summarized in [11]. The dermis and epidermis models were taken from the approximations within mcxyzn and should be used with caution if being used as part of in vivo or in vitro analysis. The generic tissue was modelled as 85% lipid from [20], 13% water, and 2% blood with 75% oxygenation (both spectra are compiled from several sources each and housed at omlc.org/spectra). Synovial fluid is mostly water, with some protein suspension and hyaluronic polymers [22]. As the protein effect on the absorption spectra is minimal, and we are not concerned with lower wavelengths where bilirubin might need to be included, we modelled the absorption as ~0.99 that of water. The hyaluronic particles are ~200 nm in size and so g~0.8 was reasonable. Like water, scatter is low, so ${µ}_{s}$ was taken as 0.1 mm^−1^. The turbid pannus is not a liquid, but a high-water content tissue with a lot of vascularization and hypoxia. As such, it was reasonable to model the absorption as 80% water with 8% blood volume at 50% oxygen saturation [23,24]. The scattering properties for this tissue were found to be similar to the surrounding tissue [3], so the same scattering model as the generic tissue was used.

While it is outside the scope of this paper to go into a comprehensive review of attempts to describe and quantify RA progression with respect to increased synovitis [25,26,27,28,29], it should be noted that results have been variable and difficult to systematize. Recent joint guidelines by the European Alliance of Associations for Rheumatology and the ultrasound special interest group Outcome Measures in Rheumatology (EULAR-OMERACT) have shifted to morphological grading [13], with grades 0–3 for healthy joints to the most advanced synovitis, and it has been found to be a valid schema for RA progression [30]. Generally though, quantitative studies at the metacarpal bare area define pathological thickening as exceeding 0.5 mm, with severe cases ranging from 2.0 mm to >4.0 mm [25] and so we selected a thickness of 1.5 mm (MC model with RA severity 100%) to represent a grade 2 pathology. This is clinically distinct from healthy tissue but prior to the gross convex distension of grade 3 disease.

The simulation modelled the RA progression in two distinct phases, the Detection Phase and the Grading Phase.

Detection Phase (0–25% Severity): Defined by a material change in the synovial cavity from clear synovial fluid (${µ}_{s}^{\prime }\;\sim \;0$ mm^−1^) to turbid pannus tissue (${µ}_{s}^{\prime }\;\sim \;1.5$ mm^−1^, ${µ}_{a}\;\sim \;0.05$ mm^−1^).

Grading Phase (25–100% Severity): Defined by the geometric expansion of the pannus layer. The pannus thickness increased linearly from 0.5 mm to 1.5 mm, and the lateral extent widened from 8 mm to 10 mm.

Table 1 and Table 2 detail the specific geometric and optical parameters used for each stage of the simulation.

For simplicity of interpretation, the epidermal layer was not modelled for the RA progression. To assess diagnostic robustness across populations, another set of models was created with differing epidermal layers. The epidermal layer properties were parameterized for three skin phenotypes based on melanosome volume fractions (f_mel_): Light (f_mel_ = 3%), Medium (f_mel_ = 15%), and Dark (f_mel_ = 35%) [11]. All simulation outputs were processed to extract two primary metrics: transmission flux and beam broadening.

For the transmission flux metric, the transmission profile, y(x), was integrated over the central 5 mm region of interest (ROI) and used as the primary grading metric ${\bar{y}}_{c}$. This 5 mm ROI was chosen because it would have less confounding from transmission behaviour in peripheral regions. To quantify beam broadening an area-based equivalent width, W, was chosen as the metric, rather than a peak-threshold metric such as the full-width half maximum (FWHM). W is defined as the area under the full transmission profile normalized by ${\bar{y}}_{c}$. This choice is useful when working with Monte-Carlo-derived profiles because it is numerically stable under discrete sampling and stochastic noise, unlike FWHM, which depends on locating half-maximum crossings. W also uses the full spatial distribution rather than being unduly influenced by just a few points in the profile. For the RA progression, reflectance flux (integrated intensity across three 5 mm-wide ROIs) was also calculated. Simulations were run until the average relative error in the ROIs was below 5%.

## 3. Results

### 3.1. Physical Validation: Probe and Phantom Performance

To establish the baseline reliability of the dual-mode optical probe, measurements were first conducted on a cohort of 13 healthy volunteers. The transmission intensity profiles exhibited a diffuse broadening characteristic of multiple scattering events in turbid media. Repeated scans demonstrated an intra-subject reproducibility within ±5%, confirming the mechanical stability of the device. The derived effective attenuation coefficient (µ_eff_) for healthy proximal interphalangeal (PIP) joints averaged 0.46 ± 0.06 mm^−1^.

A proof-of-concept measurement was subsequently performed on a subject with clinically diagnosed bilateral RA. The effective attenuation coefficient for the diseased joint was calculated to be 0.59 mm^−1^, confirming that the probe is functioning correctly; inflammatory scattering and absorption suppress optical transmission.

To further validate these in vivo findings against a controlled standard, the probe was tested on a multilayer silicone phantom doped with TiO_2_ and carbon-black pigment. The phantom results reproduced the attenuation trends observed in vivo, with measured intensities agreeing with predictions from diffusion theory to within 8% [31]. This phantom validation confirms that the signal changes observed in the subject with RA represent a physically grounded optical signature of the disease pathology and not an instrumental artifact.

### 3.2. Simulation of Disease Progression

Having validated the probe’s capacity to detect attenuation changes, we employed mcxyzn simulations to model the complete progression of RA from healthy (0%) to grade 2 (100%).

**Figure 4:**
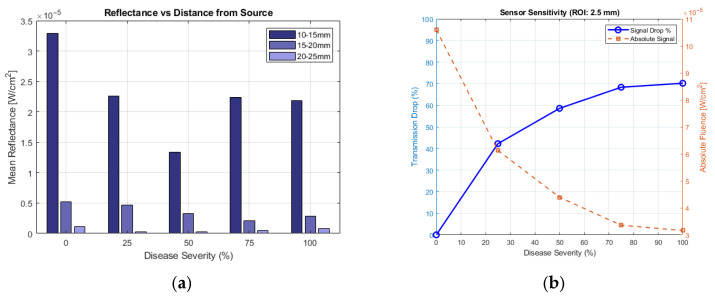
(**a**) Average Reflectance, over 5 mm intervals, for increasing severity of RA. (**b**) Transmission drop with increasing severity of RA.

The simulation revealed two distinct diagnostic regimes. In the “Detection Phase” (0–25% severity), the transition from clear synovial fluid to turbid pannus caused a sharp discontinuity in the reflectance flux for the ROI at 10 mm from the light source. This serves as a binary discriminator for disease presence, as shown in Figure 4a.

In the “Grading Phase” (25–100% severity), where the pannus geometry expands axially and radially, the transmission flux exhibited a monotonic inverse relationship with disease severity (see Figure 4b). As the pannus volume increased, the transmission signal decayed exponentially, acting as a monotonic proxy for inflammatory volume. This confirms that transmission intensity can effectively grade the extent of the hypertrophy.

### 3.3. Skin Tone Robustness and Metric Inversion

A critical finding of our simulation study is the impact of epidermal melanin on transmission-based diagnostic metrics. We modelled three distinct skin phenotypes by varying the melanosome volume fraction (f_mel_) in accordance with the established literature [11]: Light (f_mel_ = 0.03, approximating Fitzpatrick types I–II), Medium (f_mel_ = 0.15, approximating Fitzpatrick types III–IV, and Dark (f_mel_ = 0.35), approximating Fitzpatrick types V–VI). Two common metrics were assessed: transmission flux and beam broadening.

**Figure 5:**
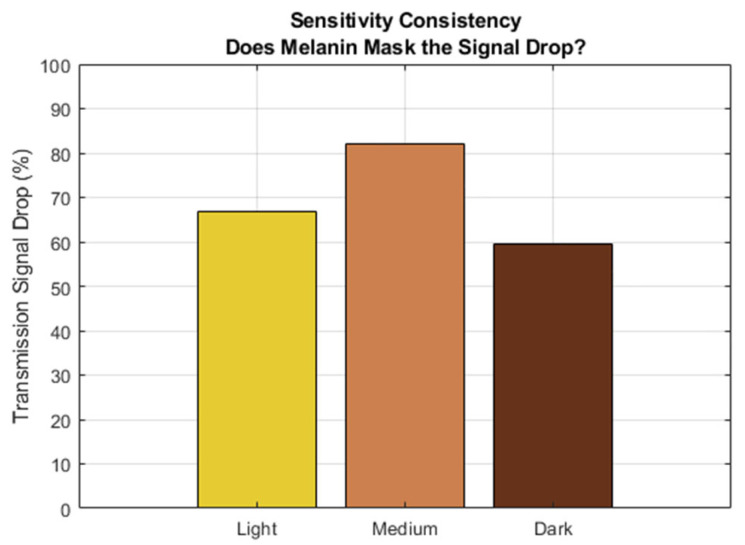
Sensitivity of RA detection across the different skin tones, defined as the ratio of ${\bar{y}}_{c}$ for 100% RA to ${\bar{y}}_{c}$ for 0% RA.

As shown in Figure 5 (Sensitivity Consistency), the transmission flux metric remained robust across all skin tones. While the absolute signal magnitude decreased significantly in Dark skin (due to high epidermal absorption), the relative sensitivity (percentage drop from 0% to 100% severity) remained stable at approximately 60–80% across all groups. This indicates that a flux-based “Volume Proxy” is clinically viable for all skin types, provided the detector gain is sufficient.

**Figure 6:**
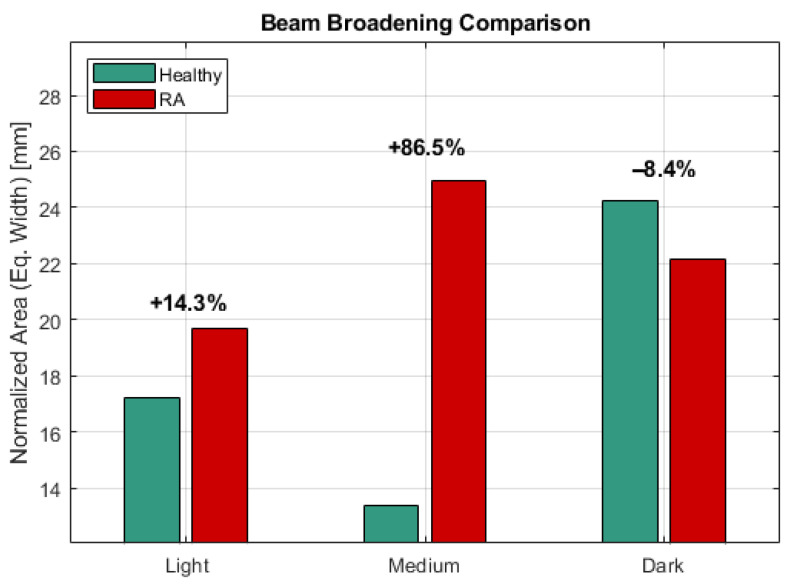
Metric inversion with skin tone.

In contrast, the beam broadening metric (often used to characterize scattering media) exhibited a “Metric Inversion” for Dark skin (Figure 6).

Light/Medium Skin: The beam width increased (+14.3% and +86.5%, respectively) in the presence of RA, consistent with increased scattering from the pannus.

Dark Skin: The beam width decreased (−8.4%) in the presence of RA.

**Figure 7:**
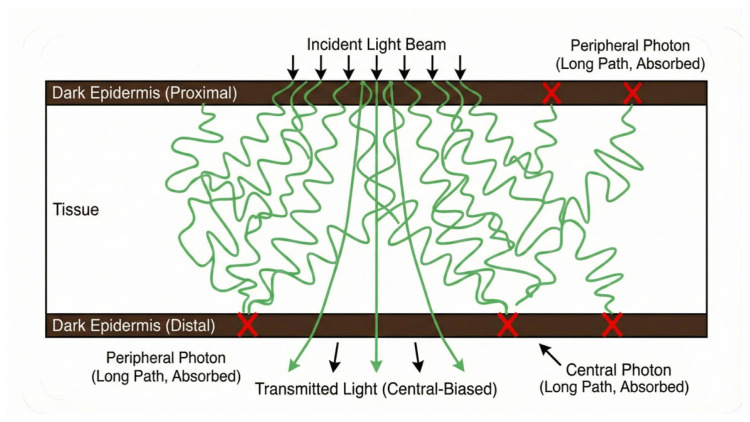
Illustration of photon extinction pathways or “pruning”. The diagram illustrates the interaction of an incident light beam with a scattering tissue volume bounded by strongly absorbing epidermal layers. Annotations highlight the absorption (red ‘X’) of both peripheral and central photon trajectories at the distal ‘Dark Epidermis’ interface, demonstrating the suppression of long path-length photons.

We surmise that this inversion occurs because the dark epidermis acts as a sufficiently strong spatial filter, or “photon pruner,” preferentially absorbing photons in the beam periphery. Those photons, which have undergone more extensive lateral scattering and thus traveled longer path lengths through the skin layers, are disproportionately suppressed compared to photons with shorter path lengths. These longer traveling photons will constitute a larger component of the peripheral regions of the exiting beam, as compared with the centre of the beam, since photons traveling shorter distances are less likely to reach the periphery (Figure 7). This differential reduction of the beam’s periphery leads to an artificial narrowing of the detected profile, even though the internal scattering has increased. Consequently, morphological metrics such as beam width may be unreliable for grading severity in Fitzpatrick V–VI populations, whereas flux-based metrics remain valid.

## 4. Discussion

### 4.1. Comparative Analysis of Geometries: Sensitivity and Dynamic Range

The results of this study suggest that reflectance and transmission geometries provide complementary diagnostic information through their distinct signal modulation characteristics. In the reflectance mode, the signal is highly sensitive to the initial transition from clear synovial fluid to turbid pannus. This is a material-driven change: the sudden introduction of scattering centers (pannus) in a previously low-scattering medium (synovial fluid, ${µ}_{s}^{\prime }\;\sim \;0$ mm^−1^) causes a dramatic increase in backscattered photon density. Even at wide source-detector separations, this reflectance signal serves as a binary discriminator for disease onset.

However, as pannus volume increases, the reflectance signal may reach dynamic range limits or saturate. In this study, the source-to-detector distance in the reflectance geometry was 15 mm. While this separation allowed for photon interrogation of deeper synovial layers, it was found to be suboptimal for the “gating” function, as it reduced sensitivity to superficial scattering events and led to early metric saturation. Future refinements to the design would benefit from decreasing this distance to improve the sharpness of the binary trigger. However, the closer the detector is placed to the source the shallower the depth that can be interrogated via reflectance. A linear array, such as we have used, mitigates this trade-off, as there are still sensor regions capturing data from deeper in the tissue. Determining a minimum source–detector distance was beyond the scope of this work due to the increasingly detailed modelling necessary for MC simulations at that scale.

In contrast to the reflectance geometry, the transmission geometry provides a volumetric proxy for disease severity by integrating attenuation over the entire joint diameter. The transmission flux decays in a predictable manner that scales with the total hypertrophic volume. This is supported by our in vivo signal verification, which showed a distinct ~28% increase in the effective attenuation coefficient (µ_eff_) in the presence of RA. Therefore, while transmission alone offers a robust measure of severity, the inclusion of a reflectance mode provides a more robust discriminator for presence detection. The dual-mode probe can address both detection and longitudinal monitoring within a single device architecture.

### 4.2. Metric Robustness and the “Dark Skin Inversion”

A critical contribution of this work is the characterization of diagnostic metric stability across skin phenotypes. While beam broadening (spatial variance) is often cited as a sensitive marker for scattering media, our simulations reveal a “Metric Inversion” for Fitzpatrick V–VI (Dark) skin types. In these cases, the high absorption of the epidermis preferentially attenuates the periphery of the scattered beam. Consequently, the beam appears narrower in the presence of disease, directly contradicting the broadening signature observed in lighter skin.

Conversely, transmission flux remained a robust metric across all modelled skin tones. Although the absolute signal magnitude decreases with melanin concentration, the relative sensitivity (percentage signal drop due to RA) remained consistent (60–80%) [Figure 5]. Therefore, to ensure clinical equity and diagnostic safety, we recommend that raw flux attenuation be used as the primary grading metric for diverse populations, while beam-shape analysis should be reserved for specific, low-absorption cohorts or corrected using more sophisticated methods, such as machine learning algorithms.

Furthermore, although not discussed in detail, our analysis indicates that morphological metrics, such as beam broadening via FWHM, are inherently sensitive to signal-to-noise ratio and post-processing filters. Unlike transmission flux, which is an integral metric robust to smoothing artifacts, beam shape parameters can vary significantly with the applied noise-suppression techniques, adding an additional layer of ambiguity to their clinical interpretation.

### 4.3. Clinical Positioning: Functional vs. Morphological Monitoring

Current gold-standard modalities, such as MRI and Ultrasound, excel at visualizing structural pathology, including bone marrow edema and synovial thickening [4,6]. However, these are morphological changes that often represent the consequence of prolonged inflammation. Near-infrared spectroscopy, by detecting changes in scattering (tissue density) and absorption (hemoglobin/water), probes the functional and hemodynamic activity of the joint. The ability of this probe to detect significant attenuation changes in a reference RA case suggests it is sensitive to the hypoxia and hypervascularity that characterize active synovitis. This offers the potential to detect hemodynamic events before they manifest as permanent structural damage. By providing a low-cost, quantitative “volume” score, this technology could fill the gap between infrequent, expensive MRI scans, enabling high-frequency monitoring of treatment response at the point of care.

### 4.4. Limitations

While the phantom and simulation results are robust, the in vivo feasibility test was limited to a single RA subject and a small healthy cohort. Furthermore, the current simulation assumes a static geometry; in clinical practice, variable finger positioning and pressure can introduce artifacts. However, the intra-subject reproducibility of <5% observed in the volunteer study suggests that the probe’s mechanical baffling effectively mitigates these motion artifacts. Notably, the signal contrast observed in the pathological case (~28% increase in ${µ}_{eff}$) was more than five times larger than the reproducibility noise floor (~5%), indicating that the diagnostic signal is statistically distinguishable from instrumental or positioning variance.

Optical diaphanoscopy and diffuse optical tomography studies of finger joints report altered absorption and scattering properties in rheumatoid arthritis compared with healthy tissue, with multiple studies indicating increased scattering and/or absorption in and around the joint cavity and statistically significant group differences in larger cohorts [1,32,33]. A 28% elevation in ${µ}_{eff}$ in a single RA subject is within the magnitude suggested by reported RA-associated changes in ${µ}_{a}$ and ${µ}_{s}^{\prime }$ (given ${µ}_{eff}$ depends on both), but whether this difference remains statistically significant as the RA cohort grows depends on the between-subject variance of ${µ}_{eff}$ and the number of independent subjects in each cohort.

While the 100% RA model is for approximating grade 2 RA severity, the axial swelling and lateral extension for this grade are approximations from other studies that have tried to map such morphological changes to clinical grading and should be taken as representative values, not definitional. Similarly, the optical modelling of the tissues is somewhat a matter of judgement, and so results derived from such models should be interpreted directionally, not absolutely.

## 5. Conclusions

We have presented the design and characterization of a dual-mode near-infrared probe for the severity grading of rheumatoid arthritis. By combining physical phantom validation with mcxyzn Monte Carlo simulations, we established that a multi-geometry approach resolves the ambiguity between disease presence and disease extent. Reflectance geometry serves as a sensitive detector for the onset of synovial changes, while transmission flux provides a monotonic, quantitative measure of pannus volume.

Crucially, our modelling identified a diagnostic issue in beam-shape metrics for dark skin tones, advocating for the use of transmission flux for severity grading. This system offers a functional, hemodynamic complement to morphological imaging, potentially enabling the detection of inflammatory flares before irreversible joint damage occurs. Future work will focus on modifying the probe geometry based on this work and expanding the clinical dataset to train machine learning classifiers capable of auto-correcting for skin tone variations.

## Figures and Tables

**Table 1:** Optical parameters used in the Monte Carlo simulations.

Material	${µ}_{a}$ [mm^−1^]	${µ}_{s}^{\prime }$ [mm^−1^]	g	n
Generic tissue	0.01145	1.288	0.90	1.37
Bone	0.00724	1.760	0.90	1.55
Synovial Fluid	0.00426	0.020	0.80	1.34
Turbid Pannus	0.04091	1.288	0.90	1.40
Epidermis (f_mel_ = 0.03)	0.3513	2.353	0.90	1.37
Epidermis (f_mel_ = 0.15)	1.744	2.353	0.90	1.37
Epidermis (f_mel_ = 0.35)	4.064	2.353	0.90	1.37

**Table 2:** Geometric parameters used in the Monte Carlo simulations to model the progression of rheumatoid arthritis from Grade 0 to Grade 2.

RA Severity [%]	Joint Layer Material	Layer Thickness [mm]	Joint Swelling [mm]	Lateral Extent [mm]
0	Synovial Fluid	0.50	0.00	8.0
25	Turbid Pannus	0.75	0.25	8.5
50	Turbid Pannus	1.00	0.50	9.0
75	Turbid Pannus	1.25	0.75	9.5
100	Turbid Pannus	1.50	1.00	10.0

## Data Availability

The datasets presented in this article are not readily available because the data are part of an ongoing study. Requests to access the datasets should be directed to Farid Golnaraghi.
